# Inferring *Plasmodium vivax* Transmission Networks from Tempo-Spatial Surveillance Data

**DOI:** 10.1371/journal.pntd.0002682

**Published:** 2014-02-06

**Authors:** Benyun Shi, Jiming Liu, Xiao-Nong Zhou, Guo-Jing Yang

**Affiliations:** 1 Department of Computer Science, Hong Kong Baptist University, Kowloon Tong, Hong Kong; 2 National Institute of Parasitic Diseases, Chinese Center for Disease Control and Prevention, Shanghai, China; 3 Key Laboratory of Parasite and Vector Biology, MOH, Shanghai, China; 4 WHO Collaborating Center for Malaria, Schistosomiasis and Filariasis, Shanghai, China; 5 Jiangsu Institute of Parasitic Diseases, Wuxi, Jiangsu, China; Oswaldo Cruz Foundation, Brazil

## Abstract

**Background:**

The transmission networks of *Plasmodium vivax* characterize how the parasite transmits from one location to another, which are informative and insightful for public health policy makers to accurately predict the patterns of its geographical spread. However, such networks are not apparent from surveillance data because *P. vivax* transmission can be affected by many factors, such as the biological characteristics of mosquitoes and the mobility of human beings. Here, we pay special attention to the problem of how to infer the underlying transmission networks of *P. vivax* based on available tempo-spatial patterns of reported cases.

**Methodology:**

We first define a spatial transmission model, which involves representing both the heterogeneous transmission potential of *P. vivax* at individual locations and the mobility of infected populations among different locations. Based on the proposed transmission model, we further introduce a recurrent neural network model to infer the transmission networks from surveillance data. Specifically, in this model, we take into account multiple real-world factors, including the length of *P. vivax* incubation period, the impact of malaria control at different locations, and the total number of imported cases.

**Principal Findings:**

We implement our proposed models by focusing on the *P. vivax* transmission among 62 towns in Yunnan province, People's Republic China, which have been experiencing high malaria transmission in the past years. By conducting scenario analysis with respect to different numbers of imported cases, we can (i) infer the underlying *P. vivax* transmission networks, (ii) estimate the number of imported cases for each individual town, and (iii) quantify the roles of individual towns in the geographical spread of *P. vivax*.

**Conclusion:**

The demonstrated models have presented a general means for inferring the underlying transmission networks from surveillance data. The inferred networks will offer new insights into how to improve the predictability of *P. vivax* transmission.

## Author Summary

The transmission of *Plasmodium vivax* has induced enormous public health problems at the global level. Natural transmission of *P. vivax* depends on interactions between anopheles mosquitoes and human beings. There are two important factors that influence its geographical spread. First, different locations may have different risks of infection due to their heterogeneous environmental and demographical profiles. Second, human mobility may bring pathogens from high-transmission locations to low-transmission locations. In view of this, to effectively and efficiently control the geographical spread of *P. vivax*, it would be desirable for us to characterize how it transmits from one location to another. To achieve this, we first build a spatial transmission model to characterize both the heterogeneous infection risks at individual locations and the underlying mobility of infected populations. By doing so, we can further infer the underlying *P. vivax* transmission networks from tempo-spatial surveillance data by using a machine learning method (i.e., based on a recurrent neural network model). Our study offers new insights into malaria surveillance and control from the viewpoint of both system modeling and machine learning.

## Introduction

As one of the malaria parasites that can infect and be transmitted by human beings, *Plasmodium vivax* has induced enormous challenges to the public health of human population. It has been estimated that 2.5 billion people all over the world are at risk of infection with this organism, among which China accounts for 19% of the global populations at risk [1]. To control, eliminate or even eradicate malaria, WHO has suggested that the most important measure is a timely response with the implementation of strategic intervention [2]. This requires the establishment of effective and efficient monitoring or surveillance systems [3]. Moreover, in practice, human mobility can introduce malaria into previously low-transmission or malaria-free areas, which has been cited amongst the significant causes of the failure of the Global Malaria Eradication Programme [4]. Therefore, it would be desirable to investigate the underlying geographical spread of malaria, which is not apparent from surveillance data. In this paper, the transmission networks of *P. vivax* characterize how the parasite transmits from one geographical location to another due to human mobility. By focusing on the malaria transmission in Yunnan province, People's Republic of China, we pay special attention to the problem of how to infer the underlying transmission networks of *P. vivax* based on tempo-spatial patterns of observed/reported cases.

Natural transmission of *P. vivax* depends on the interactions between female anopheles mosquitoes and human beings. On the one hand, the ability of mosquitoes to transmit *P. vivax* within a geographical location is dependent upon a series of biological factors, such as the daily survival rate of mosquitoes and the sporogonic cycle length of sporozoits in their bodies [5], [6]. On the other hand, human mobility between geographical locations in various temporal (e.g., daily or monthly) and spatial (e.g., intra-urban or inter-urban) scales may result in *P. vivax* transmission from high-transmission to low-transmission or malaria-free locations [7]–[9]. Generally speaking, the geographical spread of *P. vivax* has the following characteristics:

- *Complexity*. The dynamics of *P. vivax* transmission is complex because it can be affected by a large number of interactive factors (e.g., biological, environmental, and socioeconomic) at or across different scales.
- *Heterogeneity*. The geographical locations have heterogeneous transmission potential due to the dynamically-changing environments, economic development, and other factors.
- *Human reaction*. Human beings may react either passively or actively to malaria transmission at different organizational levels (e.g., governmental or individual level).
- *Sparse surveillance data*. The reported cases, especially in low-transmission areas, are both temporally and spatially sparse. For example, there are on average fewer than one reported case per 10,000 populations per year in P.R. China.

In view of this, to infer the underlying transmission networks of *P. vivax*, it would be desirable to address the following two computational issues:

- How can we model the dynamics of *P. vivax* transmission by taking into consideration the heterogeneous transmission potential caused by various factors at or across different scales?
- How can we quantify the impact of *P. vivax* transmission from one geographical location to another based on the tempo-spatial patterns of sparse surveillance data?

Mathematically speaking, the problem can be defined as follows: Let 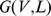 be a directed network with self-links, where 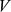 and 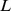 represent the sets of nodes and links, respectively. Each node 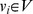 stands for a geographical location in a malaria transmission area, and each link 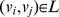 stands for the possible *P. vivax* transmission from 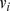 to 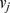. For each node 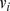, let 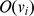 be the set of nodes that have links from 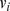, i.e., 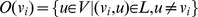, and 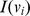 be the set of nodes that have links to 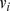, i.e., 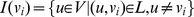. Note that 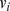 does not belong to either 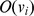 or 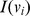. Moreover, we denote the weight of link 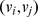 as 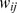 to represent the proportion of infected populations transmitting from 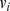 to 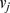. Specifically, 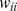 refers to the proportion of infected populations in 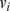 that do not transmit. In this case, the objective is to estimate the link weights of 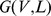 based on surveillance data, which are formulated as 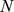 tempo-spatial series (corresponding to 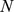 nodes or geographical locations, such as villages or towns). For each node 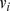, the tempo-spatial series take the form of 3-tuple 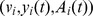, which indicates that 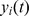 cases are observed/reported at time step 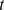 at 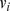 with attribute set 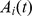. In this paper, the attribute set 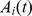 consists of the dynamically-changing temperature and rainfall over time 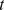 at node 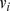, which reflects the heterogeneity of the nodes concerning the transmission potential of *P. vivax*.

**Figure 1:**
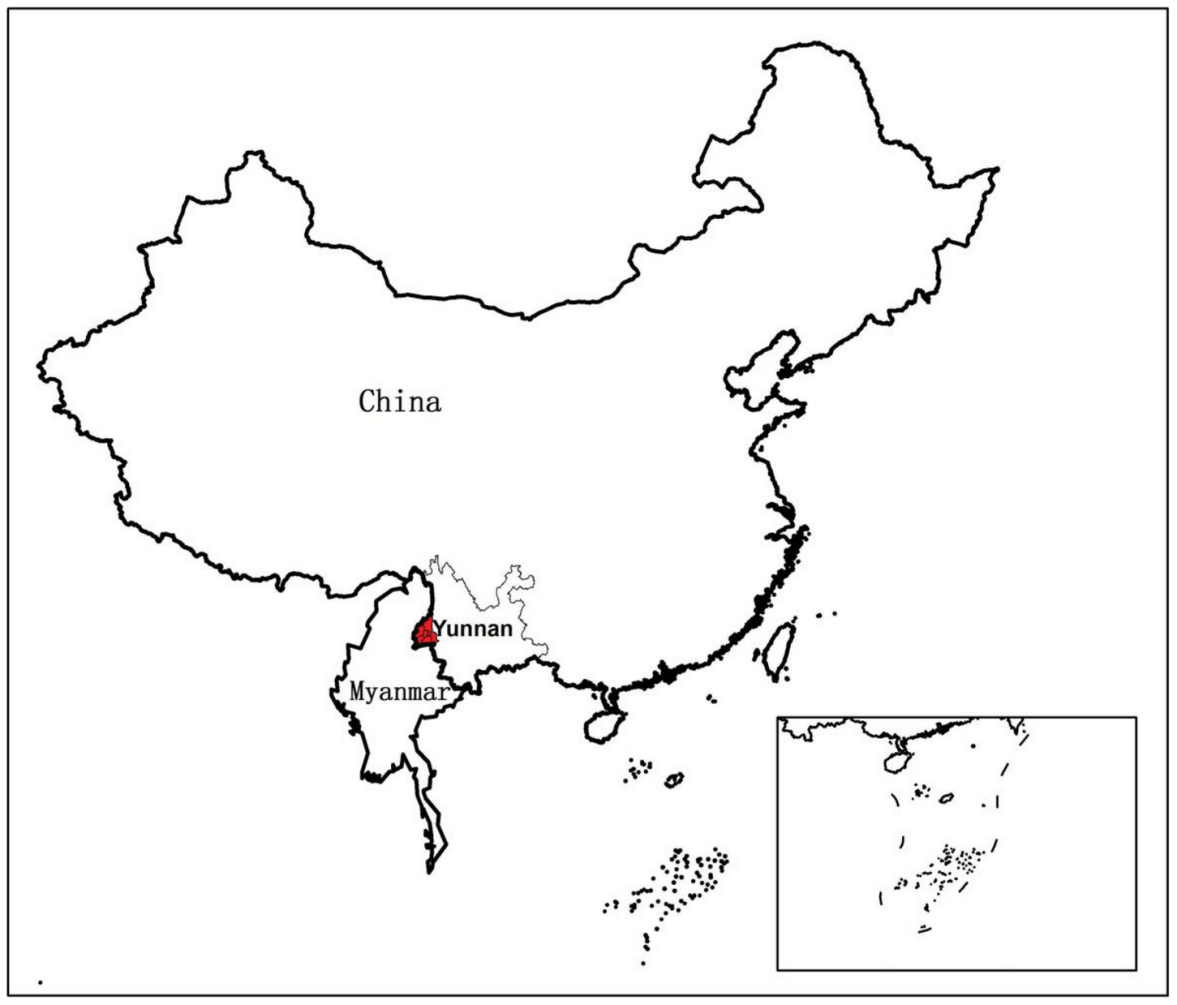
An illustration of the studied areas in Yunnan, P.R. China. The areas marked in red are located near the border between China and Myanmar.

**Figure 2:**
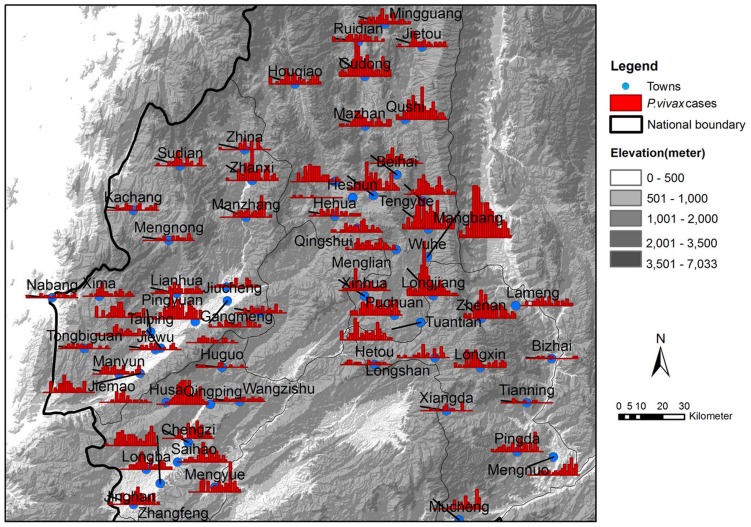
The reported *P. vivax* cases of the 62 towns in Yunnan, P.R. China, in 2005. The blue points represent the 62 towns in Yunnan. The red bars refer to the numbers of *P. vivax* cases in the corresponding towns aggregated over a duration of 16 days.

**Figure 3:**
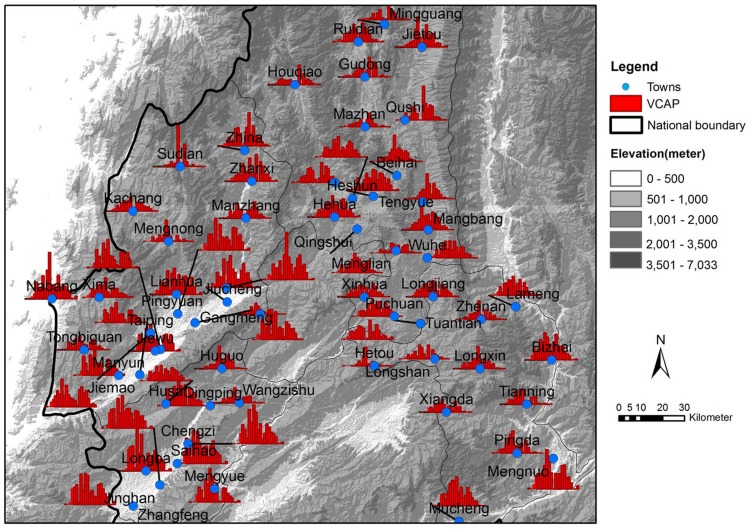
The estimated *VCAP* values of the 62 towns in Yunnan, P.R. China, in 2005. The blue points represent the 62 towns in Yunnan. The red bars refer to the estimated *VCAP* values based on the temperature and rainfall in corresponding towns and time steps (i.e., 16 days for each time step).

In this paper, we focus on the problem of how to infer the underlying transmission networks of *P. vivax* among 62 towns located in four adjacent counties (i.e., Teng Chong, Long Ling, Ying Jiang, and Long Chuan) in Yunnan, China (see Figure 1), where the IDs and names of these towns are listed in Table 1. All these towns have been experiencing high *P. vivax* transmission in the past three years, with at least one year having the annual incidence rate larger than 1/10,000. Figure 2 presents the reported *P. vivax* cases of the 62 towns in 2005 grouped by every two weeks. It can be observed that different towns has different patterns of infections. There are three major reasons: First, due to the environmental and demographical heterogeneity of these towns, the transmission potential of *P. vivax* at each individual town is different. Figure 3 shows the heterogeneous transmission potential (i.e., vectorial capacity) estimated by the average temperatures and accumulated rainfall at each town based on the method proposed by Ceccato et al. [6]. Second, human mobility from one location to another may result in geographical spread of *P. vivax*. Third, a large number of malaria cases in Yunnan are imported from Myanmar [10], which is a high-transmission country for malaria and contiguous with Yunnan.

**Table 1:** The IDs and names of the studied 62 towns in Yunnan, P.R. China.

ID	Name	ID	Name	ID	Name	ID	Name
1	Shangying	17	Mangbang	33	Mengnong	49	Mangzhan
2	Zhonghe	18	Hehua	34	Kachang	50	Nabang
3	Wuhe	19	Puchuan	35	Taiping	51	Tongbiguan
4	Menglian	20	Mazhan	36	Jiemao	52	Mengyue
5	Beihai	21	Mengnuo	37	Gangmeng	53	Chengzi
6	Heshun	22	Tianning	38	Pingyuan	54	Jiewu
7	Tuantian	23	Pingda	39	Nongzhang	55	Husa
8	Gudong	24	Mucheng	40	Zhina	56	Huguo
9	Xinhua	25	Hetou	41	Xincheng	57	Jinghan
10	Mingguang	26	Bizhai	42	Jiucheng	58	Qingping
11	Qushi	27	Lameng	43	Xima	59	Wangzishu
12	Qingshui	28	Xiangda	44	Yousongling	60	Zhangfeng
13	Houqiao	29	Zhenan	45	Zhanxi	61	Saihao
14	Ruidian	30	Longshan	46	Sudian	62	Longba
15	Jietou	31	Longxin	47	Lianhuashan		
16	Tengyue	32	Longjiang	48	Mangyun		

Imported cases in this work are defined as malaria infections whose origin can be traced to an area outside the country. Based on the annual case reporting system in P.R. China, the fraction of imported cases of *P. falciparum* in Yunnan was about 69.0% in 2005 [11]. While in 2011, among totally 301 reported *P. falciparum* cases in Yunnan, 269 of them were imported cases (i.e., the fraction of imported cases was about 89.4%) [12]. It was also reported that the fraction of imported cases of *P. vivax* in China in 2011 is about 62.9% [12]. Along this line, in this paper, we study several transmission scenarios with respect to different percentages of imported cases (i.e., 60%, 70%, 80%, and 90%) among all the reported *P. vivax* cases in the 62 towns. Specifically, we present a spatial transmission model and a recurrent neural network model to (i) infer the transmission networks of *P. vivax* from tempo-spatial surveillance data, (ii) estimate the fraction of imported cases in all reported cases for each individual town, and (iii) examine the roles of individual towns on *P. vivax* transmission.

## Materials and Methods

### A spatial transmission model

Due to the complex nature of *P. vivax* transmission, to infer the underlying transmission networks, appropriate spatial transmission model should first be formulated. In this paper, we aggregate the tempo-spatial series of surveillance data for each individual town based on a time step with duration 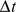. In reality, 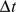 may be related to the incubation period of malaria (i.e., the period from the point of infection to the appearance of symptoms of the disease). In doing so, we assume that the observed/reported infections at time step 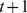 are more likely to be infected at previous time step 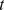. Generally speaking, the causes of geographical spread of *P. vivax* are twofold. First, within a town/node 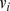, the number of malaria infections 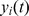 at a time step 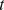 is determined by multiple factors, such as temperature, rainfall, population size, as well as the number of infections 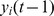 at previous time step 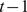. Second, human mobility may introduce *P. vivax* from one town to another. Specifically, we focus mainly on the mobility of infected populations among different towns because patients with typical malaria symptoms will be rapidly diagnosed and treated in Yunnan, P.R. China. It is seldom for a diagnosed patient to cause further malaria infection.

### Malaria transmission potential at the nodal level

To model *P. vivax* transmission at a node, we use the notion of vectorial capacity (*VCAP*), which is defined as “the number of potentially infective contacts an individual person makes, through vector population, per unit time [13].” The *VCAP* is adapted from the basic reproductive number calculated based on the Macdonald model [14]. At each node 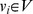, the value of *VCAP* is given by:
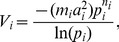
(1)where 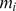 represents the equilibrium mosquito density per person, 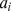 is the expected number of bites on human beings per mosquito per day, 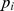 is the probability of a mosquito surviving through one whole day, and 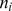 is the entomological incubation period of malaria parasites. Based on the study of Ceccato et al. [6], all these parameters are dynamically dependent on temperature (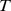) and rainfall (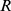) at node 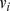. Table 2 shows the detailed parameter descriptions and settings in this work for calculating the vectorial capacity of each individual town in Yunnan. It should be noted that the values of relevant parameters are based on a certain degree of assumptions and estimates, and they could be adjusted once more accurate values are available in the future.

**Table 2:** The parameter descriptions and settings for calculating vectorial capacity.

Parameters	Descriptions	Values
**Gonotrophic cycle length**: 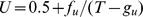		
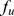	The number of degree days needed for maturation	36.5 ([6])
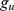	The threshold below which gonotrophic development ceases	9.9 ([6])
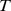	The average temperature of an individual town	MODIS ([22])
**The probability of daily survival:** 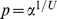		
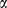	The proportion of vectors surviving each gonotrophic cycle	0.5 ([6])
**Sporogonic cycle length:** 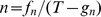		
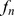	The number of degree days required for parasite development	105 ([39], [57])
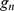	The threshold below which parasite development ceases	18°C ([6])
**Human biting habit:** 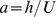		
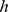	The human blood index	0.7 ([6])
**The ratio of mosquitoes to human:** 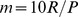		
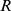	The average rainfall of an individual town	TRMM ([23])
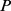	The human population in an individual town	Census ([24])

**Figure 4:**
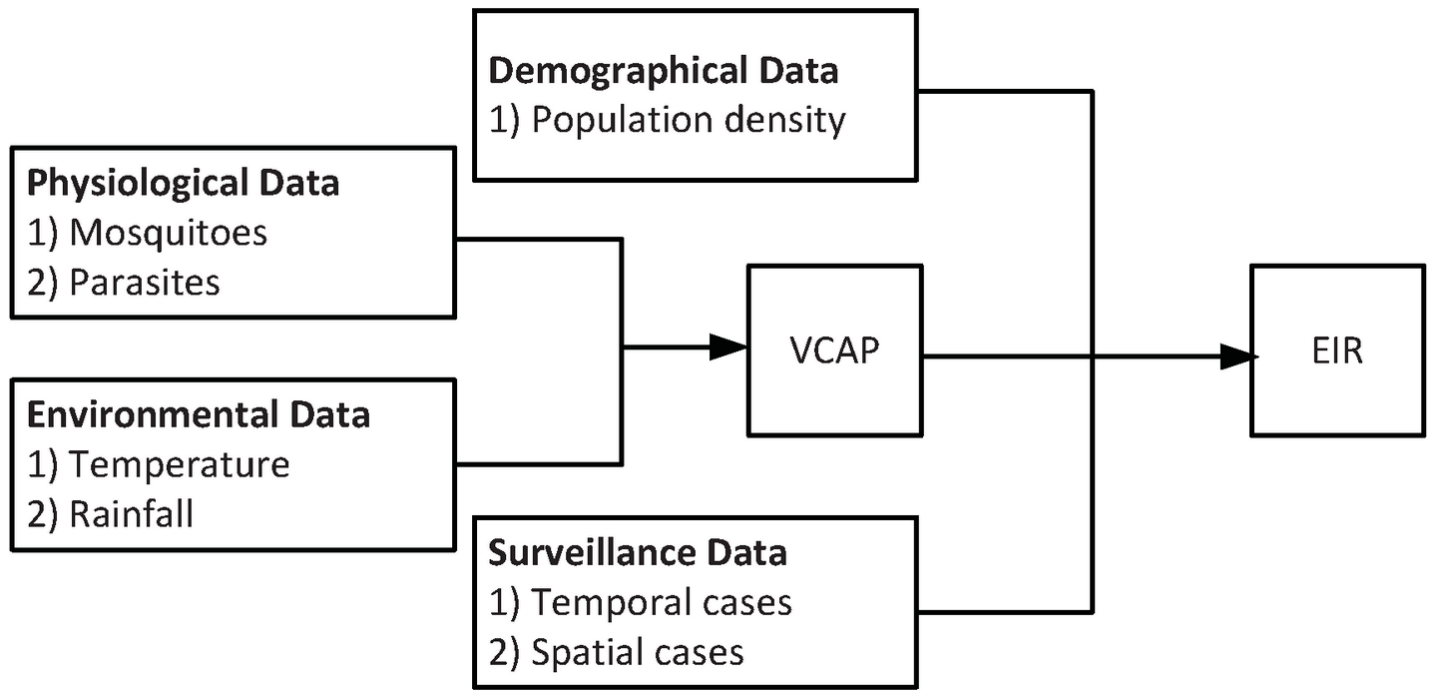
A illustration of modeling infection risks of *P. vivax* at each individual town. The notion of vectorial capacity (*VCAP*) is defined as “the number of potentially infective contacts an individual person makes, through vector population, per unit time.” The notion of entomological incubation rate (*EIR*) is defined as the number of infectious bites received per day by a human being. The calculation in this paper is based on the work of Ceccato et al. [6] and Smith and McKenzie [15].

To further estimate the number of infections at a node 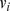, we introduce another notion of entomological incubation rate (*EIR*), which is defined as the number of infectious bites received per day by a human being [15]. Let 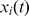 denote the proportion of infected populations among all human populations at 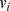 at time step 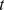, i.e., 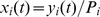. Here, 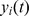 is the number of observed/reported infections at 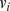 at time step 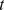, and 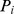 is the population size of 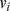. Figure 4 shows a schematic diagram illustrating various data sources utilized (i.e., physiological, environmental, demographical, and surveillance data) for characterizing the infection risks of *P. vivax* at each individual town based on the notion of *EIR*. Mathematically, 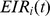 can be calculated through 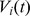 as follows:
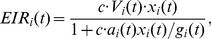
(2)where 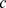 denotes the probability of the disease transmitting from an infectious person to an uninfected mosquito, 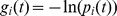 represents the daily death rate of a mosquito [15].

Based on the definition of *EIR*, the estimated number of infections without considering human mobility at time step 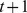 can be estimated as follows:
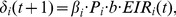
(3)where 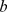 represents the probability of the disease transmitting from an infectious mosquito to an uninfected person, and 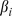 represents control impact of malaria transmission at node 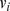. Here, the control impact 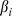 measures the efficiency of various intervention strategies implemented at node 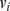, such as insecticide treated nets, and long-lasting insecticide-treated nets. Although according to Equation 3, the estimated number of human infections at 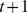 is a linear function of *EIR* at 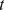, the nonlinear interactions of infected mosquitoes and susceptible human beings and vice versa are taken into account in Equations 1 and 2 associated with *VCAP* and *EIR*, respectively. Specifically in this paper, since all of the 62 towns are within Yunnan, we assume the malaria control strategies over them have the same impact. Without loss of generality, we can set 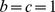, which corresponds to perfect malaria transmission between human beings and mosquitoes. In reality, these parameters can be estimated by assessing biting habits of mosquitoes at different locations and conducting virological and serological analysis on infected individuals [16]–[18].

### The mobility of infected populations at the network level

In the following, we introduce how to model the mobility of infected populations with respect to the geographical spread of *P. vivax*. Since human mobility among the 62 towns in Yunnan mainly relies on road transportation, in this paper, we assume that the transmission networks of *P. vivax* have the same *topology* (i.e., connectivity) with the transportation network. By doing so, we can quantify the transmission of *P. vivax* from one node to another by learning the link weight 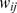 between them, which stands for the proportion of infected populations moving from 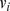 to 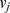 (Note that in this paper, the weight only characterizes the mobility of infected populations, where the population size of each node indirectly contributes to the weight via *EIR*). Accordingly, taking into consideration the mobility of infected populations, the number of increased infections at node 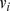 can be calculated as follows:
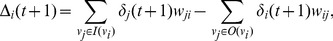
(4)which represents the difference between the number of cases transmitted from neighboring nodes and the number of cases transmitted to neighboring nodes. In summary, the estimated number of new infections of node 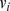 at time step 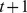 should be:
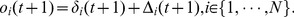
(5)


## A recurrent neural network model

**Figure 5:**
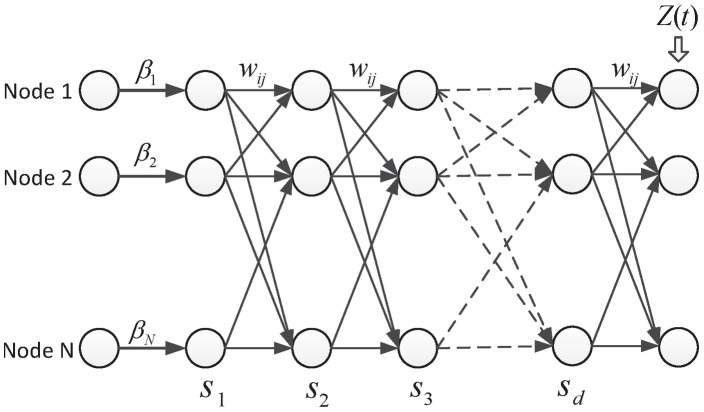
An illustration of the recurrent neural network model. There are totally 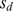 hidden layers in the neural network, each of which consists of 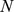 nodes representing the nodes in original 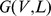. 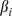 represents the control impact of each node, 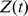 is the number of imported cases, and the links between two hidden layers are determined by the transportation network structure.

After modeling the spatial transmission of *P. vivax*, we further introduce a recurrent neural network model, which allows for reflecting both structural (or spatial) and temporal dependencies of the nodes in the network by creating interdependent internal states in the model [19]. Specifically, we build the model by taking into consideration the control impact at individual nodes, the road transportation network, as well as the total number of imported cases to the 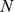 towns from the outside. Figure 5 illustrates the internal states of the model within a time step. There are totally 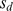 hidden layers in the network, and the links between two hidden layers are determined by the connectivity of the transportation network. Each hidden layer describes one stage of disease transmission between two neighboring towns. In doing so, to guarantee the possibility that one infected person may travel to any other towns at a time step, 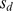 should be equal to the *diameter* of the road transportation network. The diameter of a network refers to the greatest distance between any pair of nodes in the network. To reflect the impact of *P. vivax* control at individual nodes, a vector 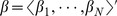 is associated to the out-links of the nodes in the input layer. In addition, the total number of imported cases (i.e., 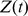) of all the 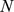 towns is linked to the 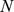 nodes in the output layer of the neural network, where a vector 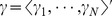 (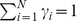) is associated with 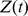 to represent the proportion of imported cases each town received in all the imported cases.

For each time step 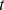, we have a vector of reported infections 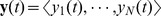, which represents the number of *P. vivax* infections at each individual town. Based on the proposed spatial transmission model, we can estimate the number of infections 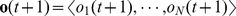 at time step 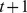 by treating 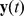 as an input. In other words, when an input pattern 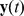 is presented to the network, it produces an output 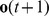, which is usually different from the number of reported cases 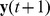 at time step 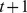. Suppose that we totally have a 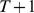 number of time steps, that is to say, we have a training set 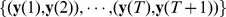 consisting of 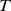 ordered pairs of 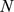 dimensional vectors (i.e., input-output patterns). In this case, the problem of inferring underlying transmission networks of *P. vivax* is to learn the parameters 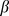, 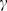, and link weights (i.e., 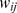) of 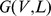 by minimizing the sum of squares of error between the estimated numbers of infections (i.e., 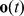) and the observed numbers of infections (i.e., 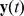) for all towns and time steps, that is,
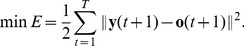
(6)To solve the problem, we can use the backpropagation algorithm. The algorithm consists of three steps: (i) feed-forward computation, (ii) backpropagation computation, and (iii) weight updates.

**Step 1:** *Feed-forward computation*. Given an initial 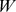 and the input vector 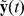, the estimated output 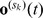 at layer 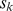 can be calculated as follows:
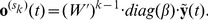
(7)Accordingly, the final output at the output layer can be calculated by
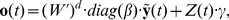
(8)where 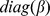 is a diagonal matrix with diagonal entries 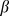.

**Step 2:** *Backpropagation computation*. The vector of backpropagation error at the output layer is computed by 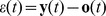. Then, the vector of *backpropagation error* at layer 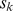 can be calculated as follows:
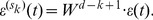
(9)


**Step 3:** *Weight updates*. After the backpropagation error has been computed for all nodes in the network, we start to update the link weights. Based on the backpropagation algorithm, the update for any link weight 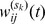 between layer 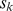 and 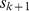 is given by:
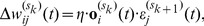
(10)where 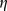 is a learning constant defining the step length of the update. Since each link has the same weight at different layers, backpropagation is performed as usual for each link and the results are simply added, i.e., 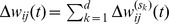. For the situation that there are 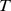 input-output patterns, the necessary update will be
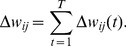
(11)The update of 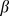 and 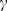 can be done in a similar way, where 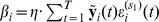 and 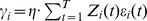.

In summary, the objective of the backpropagation algorithm is to gradually adjust the link weights so as to minimize Equation 6 by treating each time step as an input-output pattern. Theoretically speaking, the global minimum cannot be guaranteed due to the nonlinearity of the optimization problem. In this case, the step length for weight updates is set to be a small value, i.e., 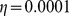. Moreover, the algorithm will be stopped when there are successive 10 times that the change of 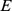 is less than 1.

## Data collection and parameter settings

The following data are involved in constructing our spatial transmission model and recurrent neural network model to infer the underlying transmission networks of *P. vivax* among 62 towns in Yunnan, P.R. China.

- *Malaria cases*. We collect the cases of *P. vivax* infection reported in 2005 from the China Information System for Disease Control and Prevention [20]. Although it is obligatory for any medical institutions and hospitals to report clinically confirmed infection cases into the system, it is ineluctable that some infection cases are under-reported [21]. While in this paper, we focus only on the *P. vivax* infections that have been reported by the system. In other words, we do not consider the possible unreported cases of the *P. vivax* infections. Specifically, we pay special attention to the geographical spread of *P. vivax* among 62 towns in four adjacent counties in Yunnan, each of which has the annual incidence rate larger than 1/10,000 for at least one year. For each reported case, we collect the infection date and location from the system.
- *Temperature and rainfall*. We collect temperature and rainfall data of Yunnan in 2005 to estimate the transmission potential of *P. vivax* for individual towns, which are located in the area between longitude ranging from 94.12134°E to 108.8718°E and latitude ranging from 20.62096°N to 29.37646°N. For the temperature, we use the Moderate Resolution Imaging Spectroradiometer (MODIS) to estimate near-surface air temperature, which are available on an 8 day basis at 1 km spatial resolution [22]. For the rainfall, we use the Tropical Rainfall Measuring Mission (TRMM) product to estimate daily precipitation, which are available on a 0.25 degree spatial resolution (about 26 km spatial resolution) [23].

Since the available MODIS and TRMM data have different spatial resolutions, we first project the TRMM data into the same resolution with MODIS data (i.e., 1 km spatial resolution). In doing so, many spatial grids may have the same values of daily precipitation. Such a deficiency can be addressed if more accurate estimates are available in the future. Then, we aggregate the daily precipitations on an 8 day basis to match the temporal resolution of the MODIS data. Finally, by respectively averaging the aggregated MODIS and TRMM data in a time duration 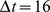, we can calculate the value of *VCAP* for each individual town based on the model proposed by Ceccato et al. [6].

**Figure 6:**
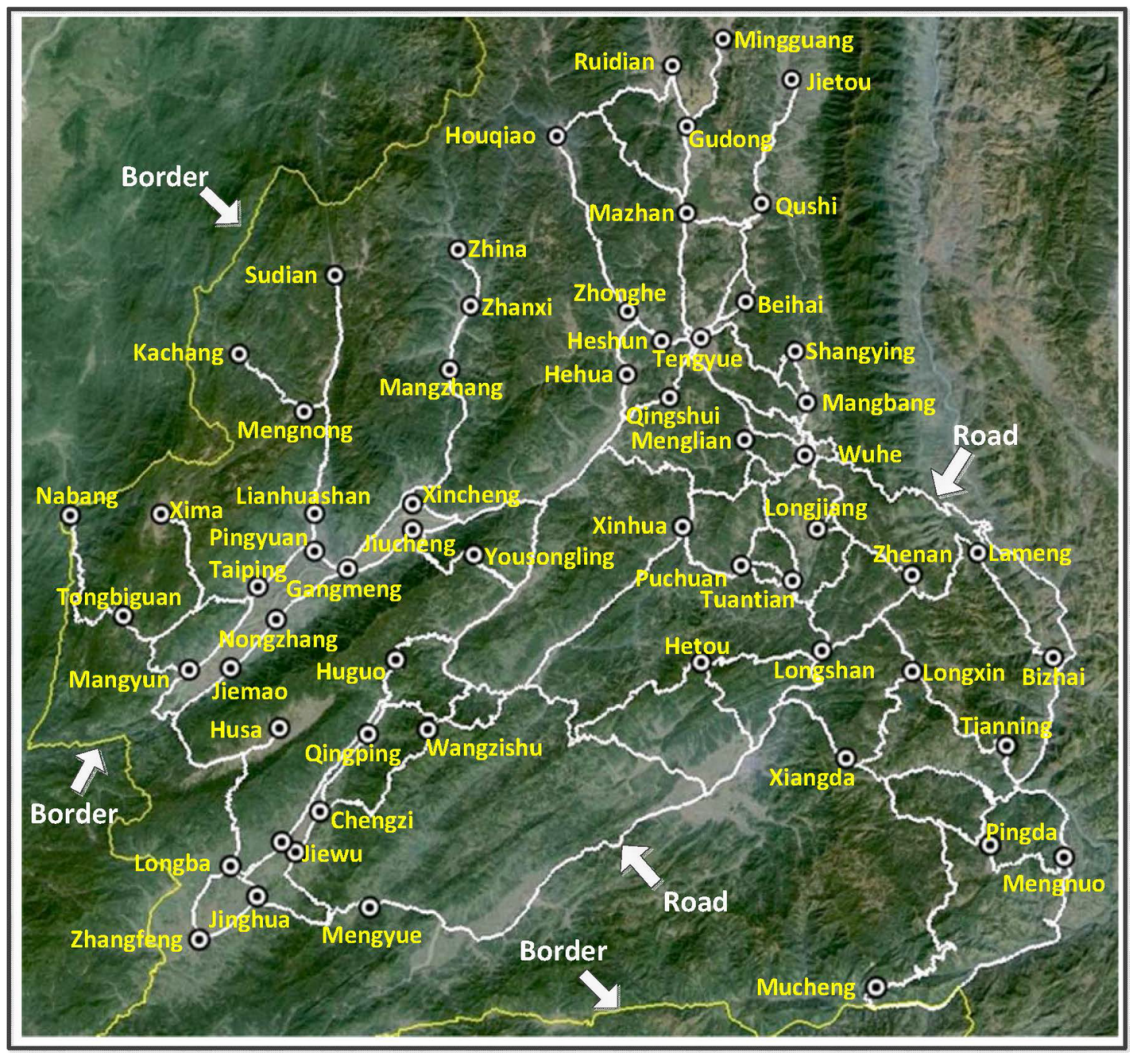
An illustration of the road transportation network among the 62 towns in Yunnan, P.R. China. The roads are obtained using Google Maps API. A direct road between two towns without passing through other towns will be included.

- *Population size*. The population size of each town is based on the national census in P.R. China. In the past decade, China conducted two national censuses, i.e., the fifth national census in 2000 and the sixth national census in 2010. However, since some administrative divisions and towns in Yunnan had been restructured after 2005, the sixth national census cannot reflect the population sizes of such towns obtained from the China Information System for Disease Control and Prevention in 2005. In this paper, we set the population size of each town based on the fifth national census in 2000 [24].
- *Time period studied*. It can be observed from surveillance data that the malaria transmission in Yunnan exhibits a seasonal pattern. In this paper, we focus mainly on the high-transmission months from April to October in 2005.
- *Duration of the time step*. Although *P. vivax* parasites may stay dormant for a long period after the primary infection is cleared from the bloodstream [25], the incubation period of *P. vivax* is usually from 12 to 20 days. In this paper, we set 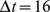 to aggregate the time series of reported cases into different time steps. There are totally 12 time steps.
- *Road transportation network*. The road transportation network among the 62 towns is identified by using Google Maps API. If there is a direct road between two towns without passing through other towns, the road between the two towns will be included. Figure 6 illustrates the identified road transportation network, where the diameter is equal to 9. In other words, we have 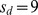.

The proposed models have presented a general way to investigate the geographical spread of *P. vivax* based on surveillance data, which involve both the heterogeneous transmission potential of *P. vivax* and a machine learning algorithm. Based on the available one-year surveillance data, the demonstrated models are able to arrive at some informative results. Accordingly, if more malaria cases are collected from surveillance data across multiple years, the accuracy of our models will be further improved.

## Results

**Figure 7:**
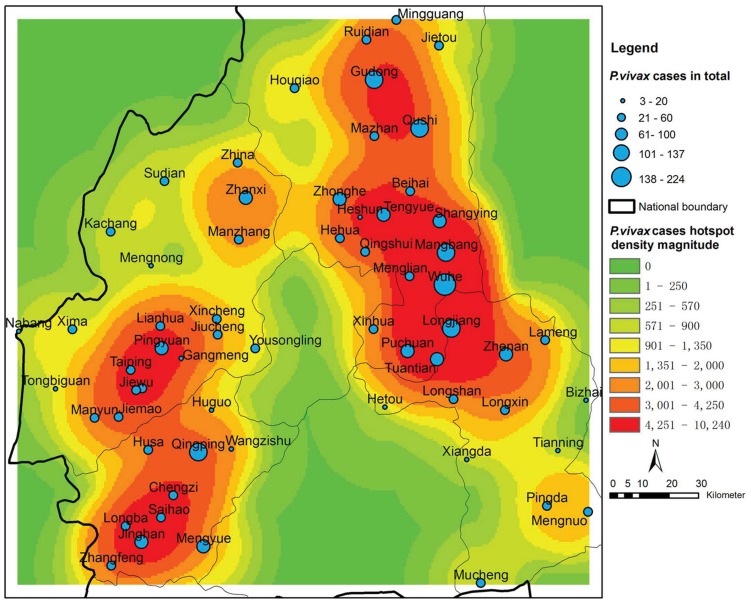
A smoothed surface map with respect to the total number of *P. vivax* cases in each individual town. The size of the nodes in blue represents the total number of reported cases. The colored surface represents the hotspot density magnitude of *P. vivax* cases after smoothing.

The number of reported *P. vivax* cases for each individual town shows a certain degree of spatial heterogeneity. Figure 7 demonstrates a smoothed surface map with respect to the number of reported cases in individual towns in Yunnan, P.R. China. The map is generated using ArcGIS version 10.0 (ESRI; Redlands, CA, USA), where the kernel density estimator with search radius 0.2 is employed. The size of a node in blue corresponds to the total number of reported cases in 2005, while the colored surface represents the hotspot density magnitude of the *P. vivax* cases after smoothing. Four obvious hotspots can be observed, that is, the areas in red around the towns of Wuhe, Gudong, Pingyuan, and Jinghan.

**Figure 8:**
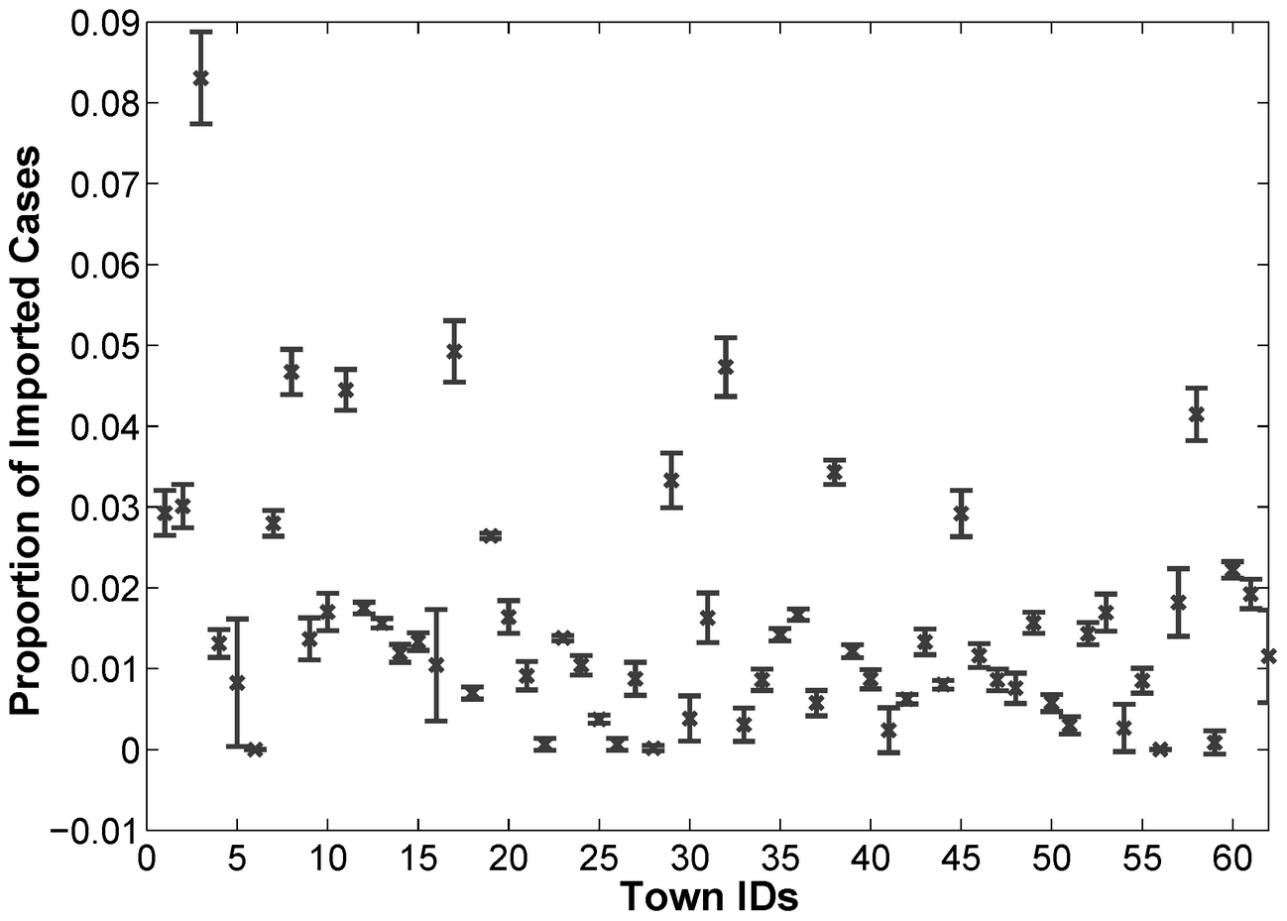
The estimated proportion of imported cases for each individual town in different scenarios. The error bars represent the standard deviations of the four scenarios with 60%, 70%, 80%, and 90% imported cases in the total number of reported cases. It can be observed that for most towns, the proportion of imported cases does not vary too much.

Based on the annual case reporting system in P.R. China over the last several years [11], [12], we assume that the fraction of imported cases among all the reported *P. vivax* cases in the 62 towns is at least 60%. Accordingly, we can estimate the proportion of imported cases for each individual town, that is, the vector 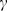 for the 62 towns. Figure 8 shows the estimated proportion of imported cases for each individual town under four scenarios with different percentage of imported cases in the total number of reported cases (i.e., 60%, 70%, 80%, and 90%). The error bars demonstrate the standard deviations, which refer to the variation of the estimated results for the four scenarios. It can be observed that for most towns, the proportion of imported cases does not vary too much. This is reasonable because international labor/tour mobility may have certain regular temporal or spatial patterns [26]. Specifically, it can also be observed that the town Wuhe has the largest proportion of imported cases among the 62 towns. This is consistent with the situation that Wuhe is the hotspot of malaria transmission (see Figure 7). From the viewpoint of active surveillance and intervention, we can pay special attention to those towns with a larger proportion of imported cases, namely, Wuhe, Tuantian, Mingguang, Tengyue, and Longjiang.

**Figure 9:**
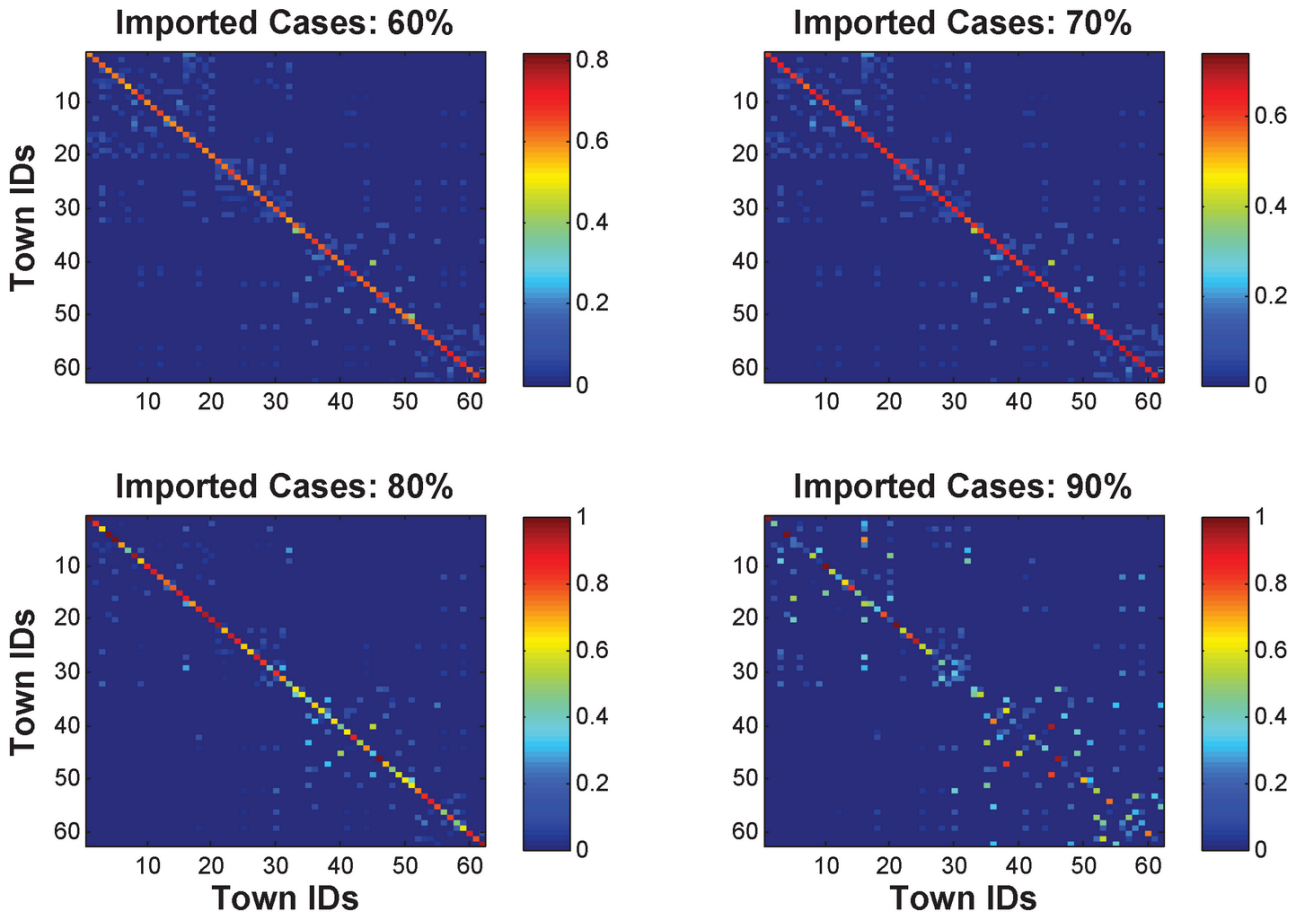
The inferred *P. vivax* transmission networks for scenarios with 60%, 70%, 80%, and 90% imported cases. The colors represent the relative strength of malaria transmission from one town to another. Note that the diagonal entries refer to the *self-propagation* of *P. vivax* within individual towns.

Figure 9 illustrates the values of weight matrices for the four scenarios with different percentages of imported cases. It seems that the inferred transmission networks of *P. vivax* (i.e., the weight matrices) show different patterns when the total percentage of imported cases changes. Particularly, it can be observed that as the total percentage of imported cases increases, the values of the diagonal entries vary dramatically. Note that the diagonal entries in a weighted matrix represent the severity of *P. vivax* transmission within individual towns (i.e., *self-propagation* of malaria) associated with their local transmission potential. This is because there is only little change about the proportion of imported cases for each individual town as shown in Figure 8. In this case, as the total percentage of imported cases increases, the total number of *P. vivax* cases caused by local infections will decrease. In other words, the *P. vivax* cases of individual towns will become geographically sparse. In this case, some towns with high malaria transmission risks may need to contribute more to the number of reported *P. vivax* cases in other towns to minimize the sum of squares for error, which makes them much easier to be identified.

**Figure 10:**
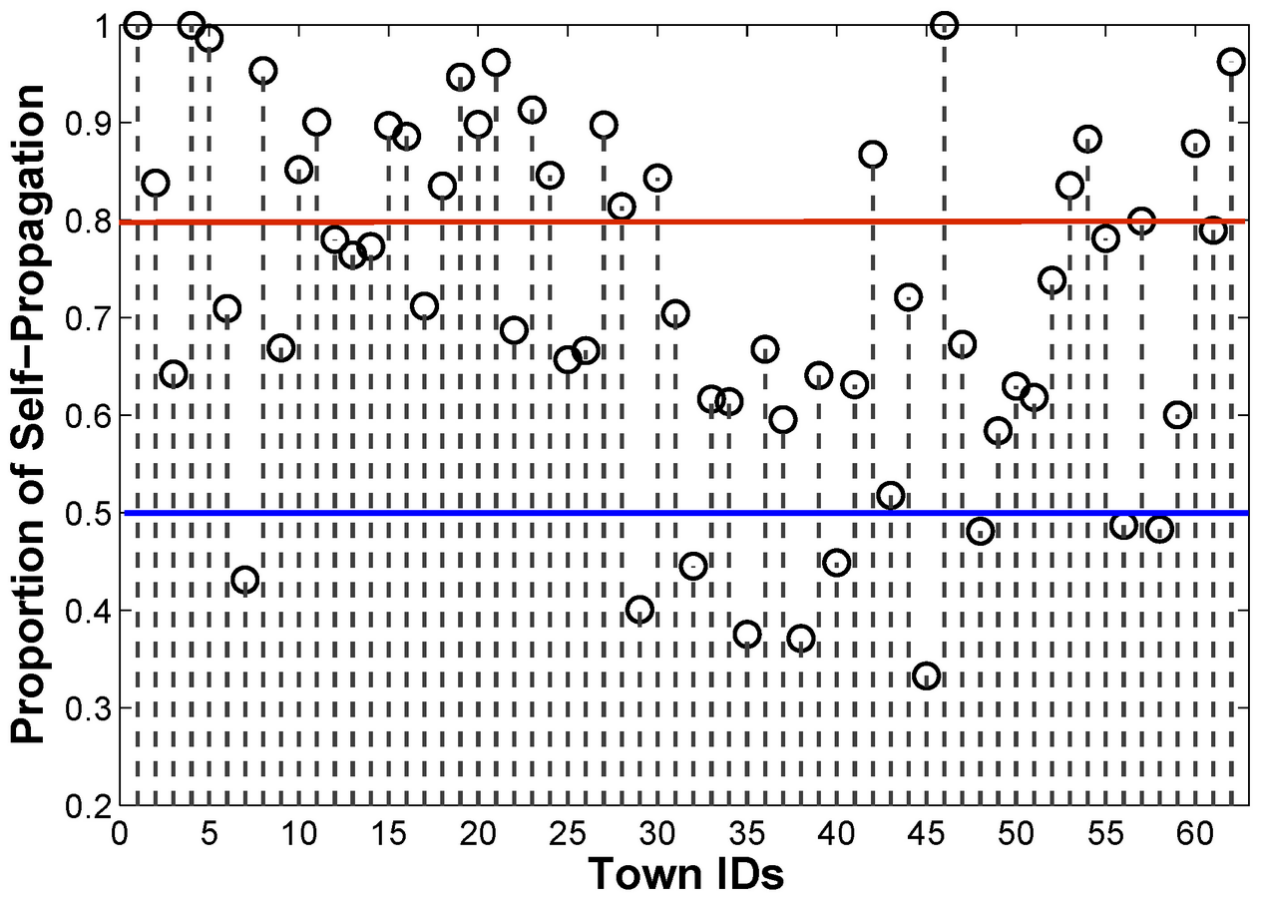
The estimated proportion of self-propagation for individual towns under the scenario with 80% imported cases. The red and blue lines show the thresholds for classifying self-propagating towns and diffusive towns, respectively.

Give the total percentage of imported cases in the 62 towns in Yunnan, we can further assess the roles of individual towns during the *P. vivax* transmission. Based on the estimated weight matrix for the scenario with 80% imported cases, the towns can be classified into two typical categories: the self-propagating towns and the diffusive towns (see Figure 10). A self-propagating town 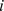 has a relatively larger 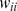, which means that fewer new infections in this town will transmit to other towns. While a diffusive town 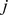 has a relatively smaller 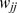, which means that new infections in this town will be more likely to transmit to other towns. Figure 10 shows an example of classification with two specific thresholds, i.e., 0.5 and 0.8. The towns with the proportion of self-propagation larger than 0.8 (respectively, less than 0.5) are classified into the category of self-propagating towns (respectively, diffusive towns). The names of the corresponding towns can be found in Table 1. In reality, the thresholds can be defined by domain experts based on their work experiences.

## Discussion

With respect to the vector-borne pathogen (i.e., malaria), existing studies have shown that human mobility from one location to another, which exhibits various spatial and temporal scales, is a key behavioral factor for its geographical spread. This is because human mobility influences their exposure to infectious vectors (i.e., mosquitoes), and further the malaria transmission [8], [27], [28]. Extensive studies have been conducted attempting to quantify human mobility patterns so as to *indirectly* predict the underlying malaria transmission networks. Such human mobility patterns can be constructed from various available data, such as survey [29], census data [30], airline transportation [31], mobile phone [9], [32], [33], or even by certain computational methods, such as the gravity model or its extension [34]. However, most of them emphasize only the impacts of human mobility, which cannot reflect the complex properties of malaria transmission. To step forward to understand the underlying transmission networks of *P. vivax*, in this paper, we have considered both the dynamics of *P. vivax* transmission and the impact of human mobility.

Another research direction focuses on understanding the critical features of host-vector-parasite interactions by building explicit mathematical models, which assume homogeneous mixing of the population [13]. Starting from the Ross model [35], a variety of differential equation models with different levels of complexity have been proposed to investigate the roles of demographic, socio-economic, and environmental factors (e.g., age, immunization, and migration), which are helpful to predict the effects of interventions on the model parameters. Along this line, to assess the effects of human mobility on the persistence of malaria, many spatial transmission models have been proposed [28], [36], [37]. One common limitation of these conceptual models is that the population of both human beings and mosquitoes are assumed to be fixed. However, researchers have shown that environmental factors (e.g., temperature and rainfall) have a significant impact on mosquito population as well as their biological cycles [38], [39]. In this paper, we have adopted the notion of vectorial capacity (*VCAP*) to characterize the heterogeneous transmission potential of *P. vivax* at different locations. Specifically, a vectorial capacity model proposed by Ceccato et al. [6] is used to monitor changing malaria transmission potential within a town by taking into consideration the impact of temperature and rainfall on the bionomics of mosquitoes and the parasite extrinsic incubation period in mosquitoes.

The last decade has witnessed a great upsurge in studying and revealing the unifying principles of real-world systems by modeling them as *complex networks* [40]–[42]. Since then, lots of efforts have been made to investigate disease transmission in populations by integrating epidemic modeling with complex networks analysis (e.g., human contact heterogeneity [43]). Each node in a network can represent either an individual or a group of individuals to model disease transmission at the individual/metapopulation level [44]. Accordingly, the transmission dynamics on the network can be formulated by stochastic models on regular networks [45] or irregular networks [46]. The mean-field versions of stochastic models on regular networks correspond to the deterministic models for which the homogeneous mixing of the population is a good approximation. One major concern of these studies is to investigate the impacts of realistic network topologies (e.g., random networks [47], small-world networks [47], [48], and scale-free networks [49]) on the process and results of disease transmission. Different from these studies, in this paper, we have focused on inferring the underlying *P. vivax* transmission based on a small-scale actual network (i.e., the road transportation network among the 62 towns in Yunnan). In the future, the proposed model may be considered for larger networks, in which a complex networks approach will be suitable.

Regarding the machine learning procedure, Liu et al. [50] have stated that the methods to infer underlying networks of disease transmission from observed incidences could be significantly different from those to infer the structures of diffusion networks from information flows due to the unique nature of disease transmission dynamics [51], [52]. Existing methods consider merely temporal information to infer diffusion networks, and most of them are based on the assumption of independent cascading of information. On the contrary, malaria may spatially propagate due to human mobility in two ways: (i) infected persons may bring the pathogen from one location to another, and (ii) susceptible persons can become infected while traveling to high-transmission locations. Therefore, geographical malaria transmission is not independent cascading. Reasonable transmission networks can be discovered only when appropriate transmission models are formulated.

As for the predictability, it is always expected that there is a powerful model that can provide accurate predictions on the malaria transmission patterns. However, it is extremely challenging due to the complicated dynamics of malaria transmission. Based on surveillance data for scenarios with various percentages of imported cases among all reported *P. vivax* cases, the hybrid model (i.e., the spatial transmission model and the recurrent neural network model) presented in this paper can help infer (i) the the proportion of imported cases for individual towns, and (ii) the transmission networks of *P. vivax* among the 62 towns. The results have shown that the proportion of imported cases for individual nodes (i.e., the value of vector 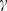) is relatively stable for different percentages of imported cases (Figure 8), while the underlying transmission networks depend heavily on the total number of imported cases (Figure 9). In P.R. China, the number of imported *P. falciparum* cases at the county level is released every year through an annual case reporting system. To further implement our models, it would be necessary to continuously monitor the imported *P. vivax* cases. By doing so, our models may provide public authorities with new insights into active surveillance and control of *P. vivax* transmission. Specifically, this can be achieved by (i) identifying whether or not a particular *P. vivax* case is imported during data collection in the front line, and (ii) analyzing the tempo-spatial patterns of imported *P. vivax* cases across multiple years.

**Figure 11:**
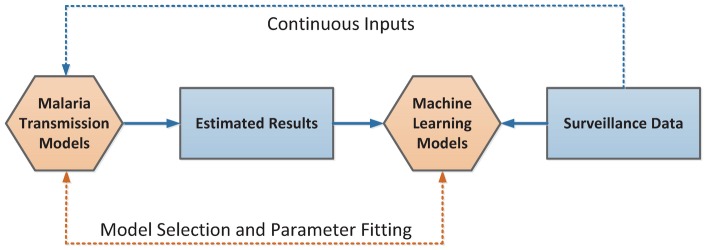
An illustration of the proposed machine learning approach to predicting the patterns of malaria transmission. On the one hand, the surveillance data can serve as continuous inputs for a malaria transmission model, which is used to predict malaria transmission patterns. On the other hand, the surveillance data can also perform as measures of an appropriate machine learning model such that both the malaria transmission model and the parameters in the model can be adjusted accordingly.

Last but not the least, this work is novel in that it provides a way to investigate the underlying malaria transmission patterns from the real-world malaria surveillance data [53], [54]. Figure 11 illustrates a machine learning framework, which consists of the interactions between malaria transmission models and machine learning models. The framework consists of three interactive components:

- *Malaria transmission models*. Based on the real-world problems that need to be investigated, appropriate transmission models can be developed ranging from conceptual homogeneous mixing models [13] to realistic data-driven agent-based models [44], [55]. Once a model is developed, some parameters should be continuously obtained from surveillance system, such as the temperature and rainfall in this work. Meanwhile, some parameters would be difficult to obtain directly from surveillance systems, which may also determine the performance of the model.
- *Machine learning models*. For the parameters that cannot be directly obtained from surveillance system, we can infer them using appropriate machine learning methods [56]. The learning process should comprehensively concern the differences between the outputs of the transmission model and the observations from surveillance systems.
- *Surveillance systems*. The functions of surveillance systems in this framework are twofold: First, the surveillance data can serve as continuous inputs for a malaria transmission model, which is used to predict malaria transmission patterns. Second, the surveillance data can also perform as measures of an appropriate machine learning model such that both the malaria transmission model and the parameters in the model can be adjusted accordingly.

The integration of the spatial transmission model and the recurrent neural network model in this paper provides a typical implementation of this framework.

Finally, due to the data availability at the moment, the proposed models still have several limitations that are worthy of being improved and investigated in the future:

- *Biological parameters*. Most of the biological parameters have been set based on the study of Ceccato et al. [6] (see Table 2). To achieve more precise prediction, specific investigation in Yunnan should be conducted. For example, the gonotrophic cycle length of mosquitoes in Yunnan may differ from that in Africa.
- *Spatial heterogeneity*. The TRMM data for daily precipitation is about 26 km spatial resolution in this paper, which is not good enough to represent the heterogeneity of daily precipitation of individual towns. Moreover, more geographical factors may be involved to reflect the spatial heterogeneity, such as elevations and vegetation of individual towns.
- *Human mobility*. This paper has only considered the mobility of infected populations among the 62 towns. By quantitatively characterizing human mobility patterns (e.g., through calling records of mobile phones [9], [32]), the results might be significantly improved. Further, for those countries/regions where human mobility from one location to another may further introduce new infections, more complex spatial transmission models should be involved into the framework [28], [36], [37].
- *Learning methods*. A recurrent neural network model is used to infer the underlying *P. vivax* transmission networks, where a time step with a duration of 16 days is utilized. In the future, novel machine learning methods will be proposed to avoid such manual settings. Moreover, to improve the accuracy of the learning results, it is necessary and desirable to continuously collect the reported cases of *P. vivax* infections every year.
- *Under-reported cases*. The performance of the proposed models in this paper depends on the quality of surveillance data (i.e., the reported cases of the *P. vivax* infections). However, in reality, the infections may be under-reported [21]. To take into account the possible under-reported infections, more deliberated models should be incorporated into the machine learning framework.
- *Imported cases*. In this paper, the proportion of the imported cases in each individual town is assumed to be constant throughout the year. In the future, it would be desirable to investigate whether this value is dynamically changing over time.
- *Dynamic transmission networks*. Similar to the imported cases, the *P. vivax* transmission among the 62 towns may also exhibit certain spatio-temporal patterns. To investigate the dynamic *P. vivax* transmission networks, it would be helpful to refine our framework by involving stochastic transmission models.
